# Health and Functional Status of Adults with Intellectual Disability Referred to the Specialist Health Care Setting: A Five-Year Experience

**DOI:** 10.1155/2011/312492

**Published:** 2011-10-27

**Authors:** L. Lee, J. Rianto, V. Raykar, H. Creasey, L. Waite, A. Berry, J. Xu, B. Chenoweth, S. Kavanagh, V. Naganathan

**Affiliations:** ^1^Centre for Education and Research on Ageing, Concord Hospital and Sydney University, Concord, NSW 2139, Australia; ^2^Developmental Assessment Service, St. George Hospital, Kogarah, NSW 2217, Australia

## Abstract

*Aims and Method.* The Developmental Disability Database in the Department of Rehabilitation Medicine at a metropolitan hospital was audited for observations on adults with Intellectual Disability living in the local region (total population 180,000) who were seen in an identified multidisciplinary specialist clinic, during 2006–2010. *Results. *There were 162 people (representing half the known number of adults with Intellectual Disability living in the region): 77 females, 85 males, age range 16–86 years. The most common complex disabilities referred to the specialists in this clinic were epilepsy (52%), challenging or changing behavior (42%) and movement disorders (34%). Early onset dementia was a feature of the group (7%). The prevalence of prescription of medications for gastro-oesophageal reflux was high (36%) and similar to the numbers of people taking psychotropic medications. The rates of chronic cardiovascular disease (2%), chronic respiratory disease (10%) and generalised arthritis (11%) were low overall, but did rise with increasing age. *Conclusions.* Complex neurological disabilities are common, and chronic medical illnesses are uncommon in adults with Intellectual Disability referred to specialist clinicians in this region. A combined, coordinated, multidisciplinary clinic model addresses some of the barriers experienced by adults with Intellectual Disability in the secondary health system.

## 1. Background

Consistent with estimates throughout developed countries, the *Australian Survey of Disability, Ageing and Carers *estimates that ~3% of the population has an intellectual disability, and for one third of that group that disability arose in childhood. Of this group who identify in the survey as having intellectual disability (or mental retardation) as a main disabling condition, ~50% are over the age of 15 years (i.e., 0.4% of the Australian population), and this ratio is increasing [1–3]. As a result of the increased longevity of people with persisting neurological disabilities [4], more interest is being taken in the health care needs of adults with Intellectual Disability. Specifically, the health policies of government agencies now encourage annual comprehensive health checks by general practitioners [5] and referral of adults with complex, or multiple, impairments associated with their Intellectual Disability, to specialist medical officers for consultations. 

 In 2006, with the express purpose of reducing some of the known barriers to good specialist health care, a specific outpatient clinic for adults with Intellectual Disability was established within the Department of Rehabilitation Medicine at Concord Hospital in Sydney, Australia. This paper reflects on the outcomes from this clinic firstly because of its novel status amongst adult rehabilitation medicine programs and secondly for the value of its data in planning for service development for this special population.

 The consultant in rehabilitation medicine in Australia is a specialist physician with training in the assessment and management of the medical and functional status of people with disabilities [6]. The assessment includes history taking and physical examination with respect to cognition, neuromotor activity, bladder function, bowel function, musculoskeletal function, organ illnesses, and behavior. The model of a multidisciplinary clinic with case conferencing is common in the rehabilitation medicine setting, for low incidence complex conditions. It provides the convenience for patients and carers to have access to a number of disciplines at one visit in comparison with attendance at multiple specialist appointments without coordination. It also allows for the convenience of discussion of benefits and disadvantages of management strategies within a multidisciplinary team at one time, in order to reach consensus on treatment recommendations. 

 In this Developmental Disability Clinic model, the patients are referred by their primary care General Practitioners to specialist rehabilitation physicians and psychiatrists who conduct their own initial assessments, and then present the cases to a multidisciplinary conference team expanded to include a neurologist, geriatrician, and designated allied health professionals. These weekly case conferences are considered the keystone of the program, offering the regular opportunity for discussion and referral for further management. These case conferences may also be attended by the relevant carers and family members, and referring General Practitioners are invited to participate by teleconference. Allied health professionals (psychologists, speech pathologists, occupational therapists, physiotherapists, dietitians, social workers, and registered nurses) from the Concord Hospital Rehabilitation Services (or the local Disability services for out-of-area patients) attend the case conferences and provide episodic clinical care when required.

## 2. Aim

The aim of this observational study was to report on the demographic characteristics, functional disabilities, and complexity of illnesses of adults with Intellectual Disability who were referred by primary care clinicians in an identified region, to medical specialists in the outpatient hospital setting in Australia.

## 3. Methodology

The research is a descriptive study based on an audit of information from a database setup in early 2006 with the approval of the Concord Hospital/Sydney University Research Ethics Committee. This Developmental Disability Database accommodates de-identified information on all attendees of the Developmental Disability Rehabilitation Clinics at Concord Hospital, and on other adult in- and out-patients with Intellectual Disability seen individually by the consultants who participate in this Clinic, at other sites throughout Sydney (rehabilitation physicians, psychiatrists, neurologists, geriatricians, and some paediatricians). 

 The data have been entered from the medical information recorded at the time of initial history taking and assessment. Outcomes of investigations and treatments are entered as they are retrieved during the episode of care. The database currently contains information on over 1400 adults with Intellectual Disability seen between 2006 and 2011.

### 3.1. Subjects

Subjects were included in this study if they had a diagnosis of Intellectual Disability and were aged 16+ years. So that the study subjects were representative of people who might be referred to this kind of clinic, subjects were only included if they lived within the four Local Government Areas (LGAs) regarded as those served by Concord Hospital (total population 180,000 in 2008). 

 The suburbs that make up these 4 LGAs are the catchment for the Aged Care Assessment Team that operates from the Department of Geriatric Medicine of the hospital. These Local Government Areas also constitute an administrative subset of the Central Sydney Division of General Practice and a subset of the government Disability Services.

### 3.2. Data

Data from the electronic database were extracted as spreadsheet files that could be analysed directly for descriptive statistics or exported into SPSS Version 18 for more detailed analysis. Data concerning hospitalisations and follow-up clinician assessments were obtained from both the database records and hospital medical records systems.

### 3.3. Measures

Data were collected on age at initial assessment, known clinician determined cause for intellectual disability, living environment, functional dependence, level of intellectual disability, medications, epilepsy characteristics, neurological disabilities, other organ disorders, lifestyle behaviours, and mental status. Subjects had their weight and height measured to determine body mass index. Note was made of challenging behaviours such as impulsive or compulsive behaviours if these activities were leading to self-injury or otherwise causing stress to carers. Carer observation on general behaviour over the previous year was recorded as “unchanged”, “fluctuating”, “deteriorating” or “much worse”. Completion of the Adaptive Behaviour in Dementia Questionnaire (ABDQ) by attending carers was also utilised occasionally by assessing clinicians to establish progress of deterioration [7].

 Information was collected on reasons for hospital admissions, details of medical interventions (such as botulinum toxin injections), and the involvement of clinicians in the twelve months prior to initial assessment.

 Data were also sought from the Disability Pension database [8] and the NSW Disability Services database [9] for pension and service recipient numbers of people living in the study region who identified as having Intellectual Disability. These data were used to estimate the proportion of people with Intellectual Disability living within the defined geographical area who were seen in the clinics. Measures used are detailed below.

#### 3.3.1. Functional Independence Measure

All subjects seen in the clinic have their functional status determined using the Functional Independence Measure (FIM) instrument [10]. The FIM is used widely in Rehabilitation Medicine settings in Australia. It is a standardised instrument whose development was initiated by a national taskforce in the USA in 1983 [11, 12]. The FIM has 18 domains of care and a seven-level scale of support ranging from “7”, independent, needing no assistance, through to “1”, completely dependent, possibly needing two helpers. 

 The 13 motor domains incorporate personal care, continence, transfers, and mobility, and there are 5 domains in the cognition group (comprehension, expression, problem solving, social interaction, and memory). A profile of scores across the domains is established for use in goal setting and review of progress. The FIM total score (FIMTOT) has been demonstrated to correlate well with hours of care need in the community [13]. 

 For this study, a person with

FIMTOT 100–126 was recorded as needing “intermittent” support (mostly supervisory),
FIMTOT 60–99 as needing “intensive” support (supervision and assistance), and
FIMTOT <60 as needing “pervasive” support (continuous assistance).

#### 3.3.2. Cause and Level of Intellectual Disability

Patients were included in the database if the carers nominated, or past files indicated, that they have had low intellectual functioning since childhood and they demonstrate the need for assistance from another person in communication, mobility, or self-care. The cause for Intellectual Disability was taken from existing files or the referring letters. When the physical examination was suggestive of an undiagnosed syndrome, and the families were agreeable, genetics testing was undertaken. 

 The descriptor of the level of Intellectual Disability has usually been applied in childhood following testing in the school setting and been retained throughout adulthood in documentation. Some people may have had cognitive neuropsychological testing in adulthood at the time of entry to employment services or accommodation support services (to establish eligibility). For many the level was an estimate made by caregivers. The descriptors follow the WHO conventions: “Mild” refers to IQ level approximating 55–69, “Moderate” refers to IQ level approximating 40–54, “Severe” refers to IQ level approximating 25–39, and “Profound” refers to IQ level approximating <25 [14].

#### 3.3.3. Comorbidities

Histories of past and current illnesses, and physical findings, were obtained from multiple sources including the subjects' General Practitioners, notes recorded in group home files, hospital medical records, or by inference from the medications, for example, hypothyroidism implied by prescription of thyroid hormone and diabetes implied by prescription of hypoglycaemics. 

 Data were also collected on whether specific management interventions were initiated as a result of the specialist clinic visits, for example, botulinum toxin injections for spasticity, percutaneous endoscopic gastrostomy feeding, behaviour management plans, epilepsy interventions, and psychotropic drug prescription.

#### 3.3.4. Hospitalisations

Hospitalisations are recorded in the files of patients attending the clinics, and note is made in the database. However, in the search for all adults with Intellectual Disability who had accessed secondary health services in the region, an additional audit was conducted for this study. The regional inpatient statistical collection (ISC) for January 2006 till December 2009, for the four public hospitals in the region, was interrogated for all separations (inpatient admissions) of adults (16+ yrs), living in the four LGAs of interest, with a comorbidity code relating to Intellectual Disability: that is, F70 Mild mental retardation, F71 Moderate mental retardation, F72 Severe mental retardation, F73 Profound mental retardation, F78 Other mental retardation, F79 Unspecified mental retardation, or F84 Pervasive developmental delay (Retts, Aspergers, Autism). This yielded 110 names. The full hospital records of these people were scrutinized to establish the correctness of the code. After excluding people whose medical records indicated a diagnosis of schizophrenia or dementia in any of these identified admissions, and confirming our inclusion criteria for the study, we established that 68 adults with Intellectual Disability had been admitted to hospital for day-only or overnight care in the period, all of whom were already known to Clinic Team members or have been followed up since that time.

## 4. Results

### 4.1. Service Recipients

In 2009/2010, there were 333 people (aged 16–64 yrs) with Intellectual Disability living in the four Local Government Areas of interest in receipt of the Disability Pension and 295 people with Intellectual Disability in the same age group in receipt of Disability Services, giving an administrative prevalence for adults with Intellectual Disability in this region, of 19/10,000. This regional prevalence is lower than expected when compared with the national survey prevalence of ~4/10,000 (80,000 15+ yrs of 19.6 M total population in 2003) [3] which would lead to an expectation of 720 adults.

### 4.2. Demographics

Information was obtained on 162 adults seen between January 2006 and December 2010, who met the criteria for inclusion in this study, that is, 162 out of probable 350 adults in the region (when an estimate for those over 65 yrs is added). Approximately 46% were referred to the specialist Intellectual Disability clinic in this time. The age range was 16–86 years at time of assessment, with a mean age of 44 years. Forty-seven per cent were over 44 years of age. Males (52%) were slightly more prevalent than females (48%). Most lived in group-home-type supported accommodation (88%). Some of the important demographic and clinical details are summarised in Table 1.

**Figure 1:**
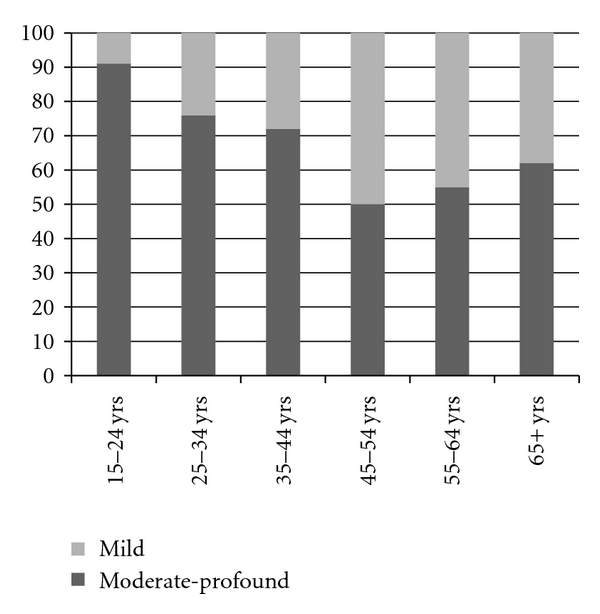
Levels of Intellectual Disability by age groups.

 Chromosomal abnormalities were identified as a cause for the childhood brain damage in 18%, and 38% had a history of a catastrophic hypoxic perinatal event. Forty-four per cent (44%) had no known cause for their Intellectual Disability. One third had mild Intellectual Disability and two thirds had moderate, severe, or profound Intellectual Disability.  Figure 1 demonstrates the variation across the age groups. 

**Figure 2:**
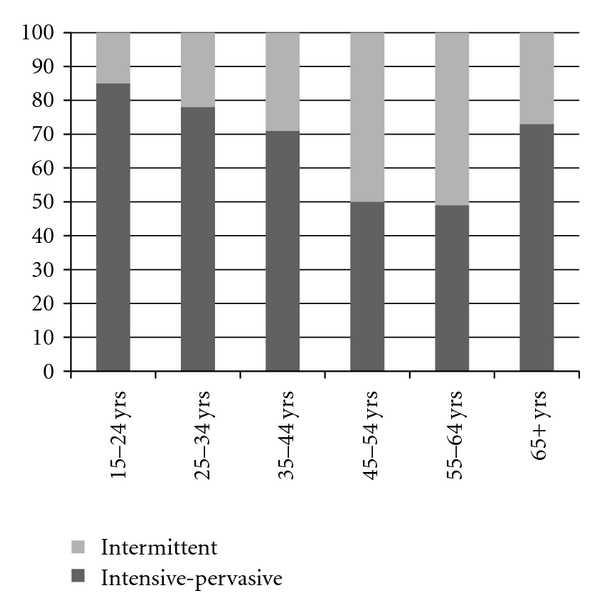
Levels of care need by age groups.

 Almost 90% of the patients in the study group were in receipt of some form of formal support. Intermittent support (drop-in or case management) was provided for 31% who lived alone or with unpaid carers who provided advice and assistance with complex executive functions such as financial planning. Twenty-three per cent (23%) needed the support of another person throughout the day for some activities of daily living. The other 46% of the group consisted of people who were extremely dependent, with FIMTOTs less than 60, i.e., they were receiving nursing-home-type assistance 24 hours per day. The largest group were those people under the age of 45 years needing intensive-pervasive support.  Figure 2 demonstrates a pattern across the age groups for dependency, which is similar to that for level of Intellectual Disability, depicted in Figure 1. 

### 4.3. Health Status and Function—Assessment and Management

Table 2 shows the lifestyle risk factors, neurological disabilities, and medical comorbidities identified at initial assessments or diagnosed by the clinicians. There was a high prevalence of neurological dysfunction (epilepsy, spasticity, and behavioural disturbance) but low prevalence of chronic disease or illness. 

Table 3 summarises the types of interventions that occurred as a result of the subjects being seen in the clinic. For twenty people (12%), the initial assessment and provision of advice to GPs was the only involvement of the Clinic practitioners. For the rest, there was a period of episodic management and followup by the relevant medical specialists, consultant nurses, or allied health professionals. 

#### 4.3.1. Lifestyle Factors

In terms of lifestyle behaviours the group had very low levels of smoking, and no cases of illicit drug use or unsafe sex practices. Thirty per cent of the group were overweight or obese, and 77% were categorized as “inactive” after questions were asked about their levels of daily activity. This level of inactivity dropped to 60% when those with movement disorders were excluded.

#### 4.3.2. Challenging Behaviour

Eighty people had challenging behaviour, specifically for which they had been referred to the Clinic. Fifty-six people (35%) were described as having some form of impulsive aggressive behaviour or compulsive self-injurious behavior. Twenty-two (13%) had demonstrated fluctuating or deteriorating behaviours in the previous 12 months. Fifteen per cent of the study group were referred, following case conference, for continuing review by the Clinic psychiatrists and/or the local Behaviour Intervention Team psychologists for special plans to assist care workers.

#### 4.3.3. Dementia

The were 22 people described as having “changing” behaviour (i.e., fluctuating or declining over the previous 12 months). Current criteria for the descriptor of “dementia” do not easily incorporate people with Intellectual Disability, and so decisions about dementia were based on the history of cognitive changes with behavioural manifestations. The Adaptive Behaviour in Dementia Questionnaire [6] was used in interviewing carers about the changes in identified behaviours over the previous 12 months. “Possible” dementia was diagnosed by the Case Conference Team in 4 people and “probable” dementia in 7. Of those 11, two people had Down Syndrome and one had Fragile X; all had onset of their dementia before the age of 60 years. 

 Carers of people with changing behaviour were offered assistance and advice in dealing with behavioural changes, and documentation was provided for five people who needed justification for more care hours in their existing accommodation environment or a shift to one in which more care could be provided.

#### 4.3.4. Spasticity

There were high rates of spasticity in the younger group. Approximately 10% of the group with significant spasticity were seen beyond their initial clinic assessments, by the Clinic rehabilitation physicians for medication management of their dystonia. Five people had botulinum toxin injections for focal spasticity in upper and/or lower limbs.

#### 4.3.5. Dysphagia

Forty-five people had dysphagia (difficulty swallowing). The Clinic physicians initiated investigations, referred to speech pathologists, and discussed with gastroenterologists when needed. Advice was offered to carers on the day to day practices which should be initiated in people with swallowing difficulty. Twenty-one people (21) had been in receipt of percutaneous endoscopic gastrostomy (PEG) feeding prior to initial assessment by this clinic. About half of this group had had the PEGs inserted as children. They continued with monitoring by their existing specialist gastroenterologists or were linked with new local practitioners. For a small number, the outcome of the clinic assessment, in relation to dysphagia, was referral to the local Palliative Care teams for ongoing management advice and conjoint care.

#### 4.3.6. Sensory Disabilities

Vision disorders (33%) and hearing impairments (12%) were also common neurological disabilities. Where relevant, nursing and allied health professionals in the clinic teams or local disability teams provided assessment and immediate management, and referrals were made to other linked specialist clinicians such as ophthalmologists when necessary.

#### 4.3.7. Epilepsy

Of the 162 people in the study, 89 had a history of epilepsy and 85 were taking anticonvulsant medications: all but 8 had childhood onset epilepsy. Of the 85 people taking anti-convulsants for epilepsy, 32 had had no seizures since childhood or were well controlled on one or two medications. Fifty three (32% of the study group) continued to experience monthly, weekly, or daily seizures.

 In accordance with the health policy operational for all residents of supported accommodation, all people taking more than one anticonvulsant were seen at least annually by a specialist neurologist and all had Emergency Epilepsy Management Plans. The study group was seen by one of four neurologists in two practice sites in the region. In two cases in the past two years prolonged admission to Concord Hospital with conjoint care by neurologist and rehabilitation physician was required. 

 Adult onset epilepsy was a new finding in 8 people of the 11 in whom dementia was eventually diagnosed.

#### 4.3.8. Chronic Illnesses

**Figure 3:**
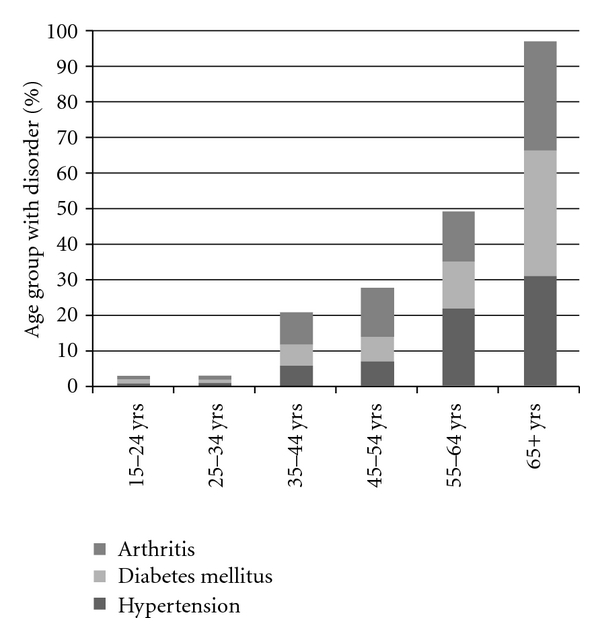
Prevalence of hypertension, diabetes, and osteoarthritis rises with age.

Prevalence of hypertension, coronary vessel illness, diabetes, chronic respiratory disorders, and osteoarthritis were low, although as is depicted in Figure 3, the prevalence of diabetes, hypertension, and osteoarthritis did rise with age. 

#### 4.3.9. Polypharmacy

Consultant physicians reviewed the physical health, investigated where appropriate and suggested changes to a medication regimen when necessary. In general, few changes were suggested to existing medications for medical illnesses, and there were few in which new diagnoses were made. Fifty per cent of the group were taking anticonvulsants, 20% antidepressants, and 26% antipsychotics as shown in Table 4. A large proportion of the study group (47%) were taking more than one class of psychotropic medication. 

### 4.4. Hospitalisations

In the four-year period, from January 2006 to December 2009, 68 of the people in the study were noted to have been hospitalised (22 of 86 aged 15–44 yrs, 20 of 50 aged 45–64 yrs, and 26 of 26 aged 65+ yrs).

 There were 77 Day-only admissions and 100 overnight stays, to any of the four hospitals in the region under study (one of which was a Centre for Mental Health).  Table 5 summarises the separations (admissions and discharges) by age groups and demonstrates an average occupancy of 60 overnight bed days per year for the whole group in any hospital in the region. When accounting for the numbers in each age group, the incidence of overnight hospitalisation was highest in the older age group.

 There were four main reasons for admission to hospital for overnight care, described in Table 6: mental health, epilepsy, rehabilitation, and miscellaneous medical disorders. In the calendar year 2009, there were 6 transfers to the Rehabilitation Ward: for recuperation following medical events (2), back pain (2), fractured neck of femur (1) and spasticity management (1). 

## 5. Discussion

This study extends our current knowledge of the health care needs of adults with Intellectual Disability by reviewing the health status of people referred by primary care practitioners to specialists in the hospital-based health system. The main findings of the study are that in this group the prevalence of significant dependency is high, the prevalence of neurological disability is high, the prevalence of chronic disease is low, although it does rise with age, and multiple specialist medical officers and other clinicians can be organized to provide coordinated care.

 Although the study group is small, it does represent about 50% of the known adults living with Intellectual Disability in a region which has a well-organized and resourced health support system. In terms of the disability support system, this region did not have a large institution in devolution in its midst, and so it is not surprising that the prevalence of adults (0.18%) may be lower than the national average of 0.4% [3].

### 5.1. Clinic Model

It is well established that people with Intellectual Disability may be disadvantaged in promoting their own health because they are unable to take this responsibility themselves, and they are unable to convey their symptoms adequately to their carers. The individual difficulties are compounded by lack of time committed by health professionals to whom they are taken, the lack of responsiveness in the secondary health system that they may experience, and the inability of carers to adequately coordinate the multiplicity of specialists to whom they may need referral [15].

 By creating an outpatient clinic service that involves multiple interested specialist clinicians, encouraging both formal and informal carer attendance, and establishing protocols with colleagues in the investigation sectors of the hospital, a multidisciplinary-clinic-with-regular-case-conferencing model can overcome some of these known barriers to provision of high-quality care. Guidelines for hospital-based specialty clinics for people with Intellectual Disability have been suggested [16], and the Concord Hospital clinic in the Department of Rehabilitation Medicine meets all of these criteria for best practice.

 In the Australian setting, the prescription of some psychotropic drugs by General Practitioners is not subsidised by the government, and their prescription is restricted even amongst specialists to psychiatrists, neurologists, and rehabilitation physicians. A large proportion of the study group (47%) were taking more than one class of psychotropic medication—a reflection of their complexity and need for specialist consultation. The Clinic setting offers the opportunity for efficient prescription and consensus discussion of the advantages and disadvantages for the patients who may benefit from this group of medications among this group of specialists. This “peer review” time is considered an important monitor of the quality of care provided by the clinic.

### 5.2. Health Status

The findings of high levels of spasticity, epilepsy, and behavioural disturbance in this group are consistent with other national and international literature on clinic cohorts and accommodation support cohorts of adults with Intellectual Disability [17–20]. The findings of age-related rises in prevalence of hypertension, arthritis, and diabetes mellitus in adults with Intellectual Disability, are also beginning to be highlighted in literature from developed countries [21–24]. 

 A level of inactivity of 60% in those who could be active (purposefully walking for 30 mins per day) is slightly higher than the figure for the general Australian population [25]. In our study, these people form the group who are overweight or obese and are being prescribed psychotropic medications. Clinic staff have been active in the development of special health promotional material for clients and carers, and in the organization of “healthy eating” and “being a healthy person” education programs.

 Although the numbers are small, our identification of early onset dementia in this group is consistent with recent studies on ageing in people with Intellectual Disability wherein it has been documented that people with Down Syndrome manifest their Alzheimer's Disease at much younger ages than the general population. Non-Down's patients may develop dementia at the same rate as the general population, or perhaps slightly earlier [26, 27]. Care and advice for the carers of this group are similar to that for people without Intellectual Disability [28].

### 5.3. Hospitalisations

About 50% of the study group had presented to an Emergency Department or been admitted to a hospital for overnight care in the past four years and their reasons for admission were numerous. Their average length of stay for overnight care was similar to that of the general population for these aggregated groupings of rehabilitation, mental health, seizure/neurology, and miscellaneous medical. While it could be postulated that people with Intellectual Disability might need a longer stay in hospital for each admission because of diagnostic overshadowing or poor communication, it appears that this is not occurring in the few hospitalizations that have been documented in our region. It could be surmised that the existence of the special clinic and its in-built followup and liaison mechanisms may be assisting in maintaining their lengths of stay at the general population averages.

 The low bed day utilization figures per year are significant in that an identified ward for people with Intellectual Disability in a regional hospital would be impractical. It is therefore even more important that organised liaison is provided to ensure that communication at all levels is facilitated and high-quality care provided.

### 5.4. Health Care Policy

Almost all of the group in the study were residents of supported accommodation, provided either by government or nongovernment organisations. The intense accountability scrutiny under which the staff are put to implement health promoting policies ensures that in general:

- the residents are living in a healthy environment,
- they have protocols in place for the early identification of risk,
- their access to interested health practitioners is facilitated.

Although all the referrals to the clinic came from general practitioners (government convention), they were often initiated by care workers in supported accommodation organisations, and many were mandated by the national prescribing rules and state Health Policy. There were low numbers of people with Mild Intellectual Disability in the study group. This is the group considered traditionally to be at greater risk of health care inequalities. It is possible that in our region they continue to be a group with significant problems that are not being addressed. It is possible also that they were being seen regularly by their General Practitioners who did not feel their needs were of a level requiring specialist care, and so they would not be visible in our specialist clinic dataset. Those who were not referred to specialist services appeared to be the younger group, and therefore possibly likely to be less complex.

## 6. Conclusions

We believe that our Clinic may be addressing a previously unmet need in our region. This study concludes that

- the known prevalence of adults with Intellectual Disability in the community is quite low (~4 in 1,000 in Australia and 19 in 10,000 in our region);
- approximately 50% of adults with Intellectual Disability in our community also have associated illnesses and multiple disabilities requiring specialist medical referral;
- almost all of that group are very dependent, and living in formal care;
- those who need specialist attention need multiple specialists' attention;
- chronic physical illness is more prevalent in the older subgroup of people referred to secondary services.

As expected, the most common reasons for referral to specialist health services for advice and treatment are related to the neurological damage or delay experienced in childhood, that is, challenging behaviour, spasticity, dysphagia and epilepsy. 

 System wide planning for increasing access of adults with Intellectual Disability to interested specialist clinicians should focus on continuing education and skill development in consultant physicians, nurses, and allied health professionals who have identified expertise in the management of epilepsy, movement disorders, neurological disability, challenging behavior, and ageing. This training and skill development may be facilitated in the setting of a multidisciplinary outpatient clinic at a regional level.

## Figures and Tables

**Table 1:** Study group characteristics.

Characteristic (Total = 162)	Frequency (% of total)
*Gender *	
female	77 (48%)
male	85 (52%)

*Age groups* (*range* 16–86 yrs)	
15–24 yrs	21 (13%)
25–34 yrs	33 (20%)
35–44 yrs	32 (20%)
45–54 yrs	28 (17%)
55–64 yrs	22 (13%)
65+	26 (16%)

*Level of intellectual disability*	
Mild (IQ 55–69)	53 (33%)
Moderate (IQ 40–54)	36 (22%)
Severe (IQ 25–39)	38 (23%)
Profound (IQ <25)	35 (21%)

*Cause of intellectual disability*	
Chromosomal abnormalities (7 Down Syndrome, 3 Fragile X)	30 (18%)
Cerebral palsy from perinatal trauma or infection	61 (38%)
Other	71 (44%)

*Living arrangements*	
At home, with no paid carers	18 (12%)
Supported accommodation (group homes, apartments, boarding houses, hostels, nursing homes)	144 (88%)

*Dependency *	
Intermittent support needed (FIM* Total 100+)	51 (31%)
Intensive support needed (FIM Total 60–99)	37 (23%)
Pervasive support needed (FIM Total <60)	74 (46%)

*FIMTOT highest score 126.

**Table 2:** Health risks and disabilities.

Characteristic (Total = 162)	Frequency (% of total)
*Lifestyle risks*	
Sedentary	125 (77%)
Underweight (Body Mass Index <18)	22 (13%)
Overweight (Body Mass Index 26–30)	32 (20%)
Obesity (Body Mass Index 31+)	17 (10%)
Smoking	10 (6%)

*Associated central nervous system disabilities*	
Active epilepsy (taking anti-convulsant drugs for epilepsy)	85 (52%)
Urinary incontinence (6 using catheters)	95 (58%)
Vision impairment (16 blind from birth, 5 cataracts)	53 (33%)
Hearing impairment (2 profoundly deaf)	20 (12%)
Dysphagia (21 on PEG feeding)	45 (28%)
Movement disorders (diplegia, quadriplegia, hemi and tri-plegia)	55 (34%)
Dementia (probable 7, possible 4)	11 (7%)

*Diagnosed medical conditions *	
Gastro-oesophageal reflux	59 (36%)
Chronic respiratory disease	17 (10%)
Hypertension	18 (11%)
Diabetes Mellitus	17 (10%)
Cardiac illness (coronary heart disease, congestive cardiac failure)	4 (2%)
Hypothyroidism	9 (5%)
Osteoarthritis	15 (9%)
Challenging behaviour (impulsive or compulsive self-injurious behaviours)	56 (35%)
Changing behaviour (fluctuating or deteriorating behaviour)	22 (13%)

**Table 3:** Specialist clinic outcomes.

Outcomes	Number of patients (% of 162)
Rehabilitation Medicine Assessment and advice only	20 (12%)
Episodic continuing specialist care:	142 (88%)
Spasticity management (rehab phys, neurologists, physiotherapists, occupational therapists, nurses)	16 (10%)
Botulinum toxin injections	5
Dysphagia management (gastroenterologists, rehab phys, speech path, dietitians, occup therapists)	45 (27%)
Behaviour management (psychiatrists, rehab physicians, psychologists, nurses, social workers)	25 (15%)
Dementia diagnosis and advice (geriatricians, rehab physicians, occupational therapists, nurses)	11 (7%)
Continence management (rehab physicians, nurses, occupational therapists)	25 (15%)
Epilepsy management (neurologists, geriatricians, occupational therapists, nurses)	80 (50%)
Psychotropic medication management (psychiatrists, physicians)	77 (48%)

**Table 4:** Medication usage.

Medications	Frequency (% of total group)
*Psychotropics*	
Anticonvulsants	81 (50%)
Antidepressants, anxiolytics	32 (20%)
Antipsychotics	42 (26%)
Movement disorder medications	8 (5%)
Multiple psychotropics (more than one form)	77 (47%)

*Anticonvulsants for epilepsy*	
One	26 (16%)
Two	22 (13%)
Three or more	33 (20%)

Antihypertensives	27 (17%)

Oral hypoglycaemics	12 (7%)

Antireflux drugs	55 (34%)

Analgesics, anti-inflammatories	43 (26%)

**Table 5:** Separations from local hospitals 2006–2009.

Age groups	Episodes	Day only days	Overnight episodes	Overnight bed days	Average length of stay for overnight stays	Overnight bed days/year
16–44 yrs (*n* = 86)	70	35	35	284	8	71
45–64 yrs (*n* = 50)	73	32	41	241	6	60
65+ yrs (*n* = 26)	34	10	24	221	9	55

Total	177	77	100	746	7	186

**Table 6:** Casemix inpatients from local hospitals 2006–2009.

Overnight bed days/year	Rehabilitation	Seizure/Neuro	Medical misc	Mental health
16–44 yrs (*n* = 86)	26	13	26	7
45–64 yrs (*n* = 50)	11	10	28	12
65+ yrs (*n* = 26)	21	6	28	0

Total	58	29	82	19
